# Health care workers’ experiences of exercise in forensic psychiatry: a qualitative focus group study

**DOI:** 10.1080/17482631.2025.2586877

**Published:** 2025-11-19

**Authors:** Henrik Bergman, Annelie Gutke, Peter Andiné, Alessio Degl’ Innocenti, Thomas Nilsson, Roland Thomeé, Helle Wijk

**Affiliations:** a Unit of Physiotherapy, Dept of Health and Rehabilitation, Institute of Neuroscience and Physiology, Sahlgrenska Academy, University of Gothenburg, Sweden; b Centre for Ethics, Law and Mental Health (CELAM), Institute of Neuroscience and Physiology, Sahlgrenska Academy, University of Gothenburg, Gothenburg, Sweden; c Department of Forensic Psychiatry, Sahlgrenska University Hospital, Region Västra Götaland, Gothenburg, Sweden; d Department of Forensic Psychiatry, National Board of Forensic Medicine, Gothenburg, Sweden; e Regionhälsan, Västra Götalandsregionen, Gothenburg, Sweden; f Institute of Health and Care Science, The Sahlgrenska Academy at Gothenburg University, Department of Architecture and Civil Engineering, Chalmers University of Technology, Gothenburg, Sweden; g Department of Quality Assurance, Sahlgrenska University Hospital, Gothenburg, Sweden

**Keywords:** Exercise, experience, forensic psychiatry, health care worker, physical activity, physiotherapy

## Abstract

**Purpose:**

This study aimed to describe the experiences of using exercise as part of treatment and rehabilitation among health care workers for inpatients in forensic psychiatric care, especially regarding the significance of exercise as part of treatment or rehabilitation.

**Methods:**

A qualitative design utilizing the focus group method, which in this case was carried out as two semi-structured focus group discussions with six participants each. Participants were health care workers from rehabilitation wards that work most closely with the patients concerning their planning and support in everyday activities. Content analysis with an inductive approach was applied using iterative close readings of transcripts.

**Results:**

Three qualitatively separate categories emerged: *Exercise is meaningful to the patients; Different professional roles related to the patients' exercise*; and *The work with patients' exercise is governed by different conditions*. There was an awareness of the positive impact of exercise on somatic and mental health among participants, and exercise was considered meaningful to the patients.

**Conclusions:**

This study contribute to the understanding of the role that health care workers could play about supporting exercise for patients with severe psychiatric illness. The participants identified organizational and intrapersonal barriers to patients' opportunities and abilities to perform physical activity and exercise.

## Introduction

Patients with psychotic disorders generally have low levels of physical activity (PA) (Pedersen et al., 2021). PA has been defined as any bodily movement that results in increased energy metabolism, while exercise is a subcategory of PA referring to regularly performed PA with the aim of improving health or physical performance (Caspersen et al., 1985). Exercise is a promising intervention for forensic psychiatry, as shown by many reports evaluating exercise in patients in both general and forensic psychiatric care (Hassan et al., 2022, Shimada et al., 2022). The forensic psychiatric care context itself presents unique challenges to exercise interventions, since in addition to the considerable time spent in inpatient care, patients are subject to physical restrictions and to considerable procedural restrictions, such as search and limitations of movement within the facility (Andreasson et al., 2014). It could be expected that patients in forensic psychiatric care have limited opportunities for spontaneous PA, making exercise interventions even more important*.* Exercise in forensic psychiatric care has predominantly been prescribed by non-experts and therefore might not be used to its full potential in this setting (Bergman et al., 2021). Health care workers (HCWs) are individuals engaged in actions with the primary intent of enhancing health. This broad category can include professionals such as physicians, nurses, physiotherapists, psychologists, and social workers. Experience from general psychiatry has shown that support from HCWs, such as nurses and mental hospital attendants increases the efficiency of exercise interventions since HCWs are working close to the patients and can support and encourage them (Beebe et al., 2011). However, little is known about HCWs’ thoughts and experiences of such interventions in forensic psychiatry.

Most patients in forensic psychiatric care have psychotic disorders (Degl' Innocenti et al., 2014). Comorbidities, such as substance use disorder, neurodevelopmental disorders, and personality disorders, are common (Palijan et al., 2009). Patients in forensic psychiatric care are more often treated with first- and second-generation antipsychotics and with combinations of several psychoactive pharmaceuticals than patients in general psychiatry (Degl' Innocenti et al., 2021). Treatment duration is considerable, with a median stay of almost 7.5 years of care (Sivak et al., 2023). All patients treated within forensic psychiatric care have criminally offended, often violently, and are admitted involuntarily to forensic psychiatric care (Svennerlind et al., 2010; Sivak et al., 2023). Research on treatment, care, and rehabilitation in the forensic psychiatric care context is scarce (Howner et al., 2018, Howner et al., 2019). However, patients with psychotic disorders show an over-representation of multiple somatic disorders such as diabetes, neurological disease, ischemic heart disease and stroke (Jackson et al., 2020, Launders et al., 2022) compared to the general population. Thus, psychotic patients have a shorter life expectancy compared to the general population (Sariaslan et al., 2022), and an even shorter life expectancy has been reported for those in forensic psychiatric care (Fazel et al., 2016).

Physical activity is an important protection against several types of somatic disorders (Kazemi et al., 2024; Reddigan et al., 2011). Although exercise has been shown to have positive effects on depression (Schuch et al., 2016), and cognitive function in schizophrenia (Dauwan et al., 2016, Firth et al., 2017, Stubbs et al., 2018), as well as a possibly reduced desire for drugs (Zschucke et al., 2012), patients with psychotic disorders in general psychiatric care commonly have low levels of PA (Stubbs et al., 2016), low maximal oxygen uptake (Vancampfort et al., 2017), and impaired muscle strength (Nygard et al., 2019). An increased use of exercise in psychiatry has been recommended by the European Psychiatric Association (Schuch et al., 2016) and the International Organization of Physical Therapists in Mental Health (Stubbs et al., 2018). Varied levels of PA and low levels of maximal oxygen uptake have been found in patients in forensic psychiatric care (Bergman H et al., 2020), and exercise has been suggested for this patient group (Andine & Bergman, 2019). The positive effects of PA have been highlighted in the literature (Wynaden et al., 2012), along with research mainly in older adults (Toots et al., 2016) showing the benefits of performing PA outdoors compared to indoors. Ulrich et al. (2018), explained that patients treated within forensic psychiatric care can benefit from actions meant to lower their stress levels and thereby diminish threats and violence (Ulrich et al., 2018). How to best implement exercise as an intervention in forensic psychiatric care remains an open question, however.

There are no general guidelines for the use of exercise in forensic psychiatric care and practice may therefore differ between HCWs. Support from HCWs has positively affected exercise participation for patients with psychotic disorders in general psychiatry (Beebe et al., 2011; Mason & Holt, 2012). Effects of (Stubbs et al., 2018) and adherence to exercise interventions (Vancampfort et al., 2016) increase if interventions are supervised by qualified experts (Stubbs et al., 2018), such as physiotherapists. Understanding HCWs’ perspectives on exercise, and how it transfers into individual support regarding patient and context, is therefore of importance.

In earlier studies, nurses reported viewing exercise as a part of an ideal, holistic health approach to improve both physical and mental health for patients with mental illness. However, barriers to exercise were present that contributed to fragmentation of this ideal holistic approach (Happell et al., 2013), even though most nurses in psychiatric care have prescribed exercise to patients with mental illness (Stanton et al., 2015a, 2015b). In one study, nurses agreed that there were intrapersonal barriers to exercise participation but disagreed on whether organizational barriers prevented exercise prescription by nurses. In contrast, although HCWs reported believing exercise to be beneficial to patients, they also reported that organizational and personal barriers limited their efforts to promote exercise in the forensic psychiatric care context (Kinnafick et al., 2018). Taken together, the known benefits of exercising could improve both somatic and mental health as well as cognitive function in patients within forensic psychiatric care. In order to implement exercises, we need to better understand the HCWs' experience including their thoughts and notions concerning the use of exercise in forensic psychiatric care. The rationale for this study lies in the limited understanding of how HCWs perceive and implement exercise interventions in forensic psychiatric care, despite their known benefits for patients with psychotic disorders.

This study aimed to describe the experiences among HCWs concerning the use of exercise as part of the treatment and rehabilitation for inpatients in forensic psychiatric care, especially regarding the significance of exercise as part of treatment or rehabilitation.

## Materials and methods

### Study design

This study utilized the focus group methodology since it emphasizes the interaction and co-construction of meaning among participants (Kitzinger, 1995; Krueger, 2015).

Two semi-structured focus group discussions were conducted with six participants in each.

#### Setting and participants

HCWs were recruited at a forensic psychiatric university hospital in Sweden. The hospital consisted of six wards, of which four were of medium security, i.e., rehabilitation wards, and two were high security wards. The hospital, which is one of the largest forensic psychiatric hospitals in the country, had beds for about 100 inpatients.

Each patient has a so-called contact person, who plans and supports everyday activities. They are the HCWs who work most closely with the patients and are therefore likely to have the best opportunities to influence patients’ PA and exercise habits, especially in the absence of formal routines regarding the use of exercise. Contact persons are a heterogeneous group and may have bachelor’s degrees in subjects such as social care, pedagogics, or mental health care or work as auxiliary nurses or mental hospital attendants. There are no registered nurses among HCWs. All study participants were recruited from HCWs working at the rehabilitation wards, where patients spend most of their time as inpatients. The rehabilitation wards, to a larger extent than the high security wards, have access to hospital exercise facilities, such as the gym, a hall suitable for racquet and team sports, and a pool.

The inclusion criteria for the study participants were that they should:

a) work in a rehabilitation ward at the actual forensic psychiatric university hospital.
b) have the role of being a contact person for at least one patient.

One of the study researchers visited regular staff meetings on all four rehabilitation wards to provide oral and written information about the study. Next, the head of each rehabilitation ward informed staff that participation was voluntary, but did not take further part in the recruiting process. A researcher (HB) encouraged variation among potential participants regarding age, sex, number of years of employment, education, own habits regarding PA and exercise, and presumed opinions on the use of exercise in care. Potential participants were formally asked to participate by the researcher and written informed consent was obtained.

The study was approved by the Swedish regional Ethical Review Authority in Gothenburg (Ref. 242−16).

#### Data collection

Rather than conducting individual interviews, study participants were divided into two focus groups of six participants each to allow between-subject interactions (Kitzinger, 1995; Krueger, 2015). In groups of people who share common experiences, participants are more likely to exchange thoughts and experiences (Kitzinger, 1995). The group’s common reflections take advantage of the group dynamics when participants ask questions and give comments on each other’s thoughts (Kitzinger, 1995; Krueger, 2015).

Focus groups were moderated 1^st^ and 5^th^ of September 2016 at the hospital by an experienced nurse (PhD, female) (HW). A physiotherapist (PhD, female) (AG) was present as an observer. Neither the moderator nor the observer was known by the participants or worked clinically at the forensic psychiatric hospital. The moderator led the discussion, initiated conversation topics, and ensured that all participants were given the opportunity to participate. Focus groups were semi-structured and open-ended questions were used. A pilot-tested interview guide covering the subjects of interest was used as a support for the moderator. Questions were not asked in a specific order, and follow-up questions were used to deepen the understanding. The discussions took place in a secluded setting adjacent to the participants' workplaces. The two focus group sessions were 57 and 62 minutes long, respectively. The focus group discussions were recorded, transcribed verbatim by HB, and the text served as the unit of analysis. Additionally, the observer took notes on any interactions that took place. Before data processing, all data were pseudonymized with letter codes, and only HB had access to the code key.

#### Qualitative analysis

Content analysis with an inductive approach was chosen for the analysis of the data (Graneheim & Lundman, 2004, Graneheim et al., 2017). The analysis was data-driven rather than theory-driven. The focus was on manifest content, however, since the analysis of manifest content involves interpretation, the level of abstraction varied in relation to the analysis of latent content. The material was first read several times to get a general view of the content. During the analysis, the text was reread several times to ensure a comprehensive understanding. HB initiated the analysis with support from HW. Meaningful units, the parts of the data relevant to the subject of interest, were identified, condensed without losing their message, and assigned a code number to maintain the possibility to back-track with the original interview transcript. Codes were sorted on their similarities and then abstracted and interpreted into subcategories. AG, who is unfamiliar with the forensic psychiatric care context, participated in the recontextualization phase, comparing the codes and how they were sorted into subcategories. In the next step, subcategories were sorted based on their similarities and then abstracted and interpreted into overarching categories. The coding and categorization were continually discussed with all authors until consensus was reached.

### Preunderstanding

To facilitate a broad understanding, the research group consisted of seven researchers, *two females and five males* with different professions, competencies and experiences from both clinical work and research areas. HB was a physical therapist and PhD student with 25 years of clinical experience who had worked within forensic psychiatric rehabilitation, where the data collection started in 2011. To mitigate potential bias, HB did not participate in the data collection. HW is a professor of nursing science with extensive experience in *qualitative research methodologies.* AG, an associate professor with 30 years of clinical experience and research in physiotherapy, and RT professor in physiotherapy with extensive experience in clinical research of sports medicine, focused on physical activity and exercises in this project. Data collection was conducted solely by HW and AG, who had no prior contact with the participants. PA, a medical doctor and professor in forensic psychiatry with more than 30 years of clinical work and research within forensic care, covered the medical perspective. TN professor in forensic psychiatry, directed on clinical psychology, and AD associate professor in psychology and senior lecturer in forensic psychiatryboth psychologists monitored the psychological aspects of the study.

All authors, representing diverse competences and research fields of relevance for this study, participated in the discussion of the results until consensus was reached. This reflexive approach acknowledges the researchers’ professional roles and experiences, ensuring transparency in the integration of data while addressing potential influences of preunderstanding on the research process (Graneheim et al., 2017).

## Results

Results were arranged under three qualitatively separated categories: 1, Exercise is meaningful to the patients; 2, Different professional roles related to the patients' exercise; and 3, The work with patients' exercise is governed by different conditions. Each of these categories was further divided into subcategories (Table I).

**Table I.** Citations (C1–C14) from the focus groups discussions.

C1	*… and then you see how the patient is so happy when they come back after that activity*
C2	*But the advantages of physical activity are also that you will naturally take care of hygiene, showering and the like. It is easy to point this out to the patient when you have exercised*
C3	*… It reduces the tension. And that means that my tension also goes down, and I get better in my treatment of the other patients as well (…) I also get better in my professional role if I can meet the patient's need to move*
C4	*Find out, what are they interested in. What would they like to try, maybe?*
C5	*If we do not address physical health, the doctor will not do it, and neither will the social worker and probably not the occupational therapist or the psychologist*
C6	*Many of our patients have had a very close contact with the physiotherapist (…) Then once exercise has become a habit, they can hand it over to the staff*
C7	*I know that my patient has various disabilities. He has to be prepared maybe an hour before he goes to the pool, so I go and knock and wake him up (…) it might be various actions like this that you do as a contact person.*
C8	*It is for the patient good or bad luck who you have as a contact person, or nurse then. How engaged or interested they are*
C9	*We have a new patient who gets a little unruly but as soon as we offer an activity, like going out and kick some ball, he then comes back in and is calm at the ward. So, we will use that method quite a lot*
C10	*(…) you do not feel well. Anxiety, worry, a lot of delusions or various symptoms as well, which puts sticks in the wheel for the active rehabilitation*
C11	*There is a large smorgasbord here of various physical activities. I myself am involved in the gardening group and there is also some physical activity there (…) chopping wood and digging and things like this (…) it suits some who may not be interested in anything else*
C12	*Maybe that they would like to go out, a few patients. But then we are too few staff (…) And then there will be maybe three left in the ward, and then you do not have the opportunity to keep this with security either*
C13	*That all patients must change clothes when they have been outside the hospital building, even with staff. Change completely naked and get new clothes. (…). And then there are patients who choose not to go out at all. So, it's an obstacle*
C14	*I would like it to be seen why it is good in the treatment plan. So that exercise gains some status.*

1. Exercise is meaningful to the patients

Participants described exercise as significant and effective, with different consequences for the patients. Two subcategories were distinguished within this category.

1.1 Exercise is meaningful for patients' well-being

HCWs reported that patients who exercised increased their well-being, mainly in mental and psychological areas. Patients felt better, got happier, experienced improved sleep and circadian rhythm, and the use of sleeping pills could be reduced, if they exercised.

*(C1)*

However, participants shared that exercise cannot be used indiscriminately without risk. In some cases, exercise may contribute to the worsening of the patient's condition. Patients who are on their way to be, or are in a manic state, may need to be restricted. Otherwise, they might want to try everything that is suggested so that the manic state would be aggravated. There were conflicting opinions among participants as to whether exercise was appropriate when a patient's physical ability was very low. There was a fear that exercise could provoke heart disease in some patients as their physical ability was so low that even a very low-intensity daily activity was strenuous. In contrast, it was also described that exercise can protect against a heart attack and that inactivity was the bigger risk. Furthermore, participants described that many patients were overweight and there was a risk of injurious falls, for example when participating in team sports.

1.2 The social content of exercise is meaningful for everyday life and relationships

Participants emphasized that it was the social event that was important for some patients, rather than the physiological effects of exercise. Exercise was an activity that could be suitable for a patient to perform outside the hospital in a commercial gym. Thus, exercise could be good for so-called “social training”, i.e., it became a way to practice and apply skills needed in daily life after discharge. Examples of such functions were the use of public transport or maintenance of personal hygiene.

*(C2)*

Furthermore, participants described that the social content of the exercise situation can be of particular importance for the development of a trusting relationship between them and the patient. When performing an exercise activity, such as a walk together, the conversations flowed more easily and naturally. Exercise could also have a stress-reducing effect on both the patient and the HCW.

*(C3)*

Risk situations related to the social aspect of exercise were also described by the participants. For example, exercise equipment could be used as a weapon and increase the risk of violence. Furthermore, patients may feel overwhelmed and exposed when changing clothes before or after exercise. Participants described that some patients experienced it as stressful to be in a public dressing room.

2. Different professional roles related to the patients' exercise

This category contains four subcategories (Table I). To support and facilitate the patients' exercise can be described as part of the participants’ designated tasks. Participants described various roles they could assume as contact persons, which are important for the patients' exercise. These roles can be related to the patient or to other professions within interprofessional collaborations. Participants also described that one’s own preference plays a role in patients' exercise.

2.1 To contribute with knowledge of the patients' individual conditions and draw attention to patients’ need for exercise

Participants described that they, as contact persons, could learn about specific patients’ interests regarding exercise, including which types of exercise among those available at the hospital, the patient would like to try.

*(C4)*

With their knowledge of the patient's condition, participants contributed to the treatment planning for the patient. Participants described a risk that the patient's need for exercise would not be discussed at all unless the contact persons did so.

*(C5)*

2.2 To collaborate with other categories of HCWs

Participants (contact persons) described collaborations regarding exercise with other categories of HCWs, such as physiotherapists and occupational therapists. For example, a physiotherapist could design and initiate an individual exercise program, after which the responsibility for supporting the patient was handed over to the contact person.

*(C6)*

Furthermore, contact persons described other categories of HCW such as health educators, as contributing to the inclusion of exercise in discussions of treatment plans at team conferences.

2.3 To support and motivate the patients

Participants felt that they could play a decisive role in influencing patients’ PA. This included compensating for patients’ impaired cognitive abilities by acting as a cognitive support, or helping to motivate patients when the patient's own motivation was low.

*(C7)*

Participants described that it was not formally regulated if and in what way it was part of the contact persons' tasks to support the patients’ exercise. Instead, it was the contact persons’ own thoughts and preferences about PA that were governing their actions. Moreover, participants described the individual assigned contact person as decisive for whether the patient received exercise support. Each contact person's personal experience of exercise, such as their own sports background or interest in exercise, was described as particularly important both for whether patients got to exercise and how well the contact person managed to support the patient in exercise.

*(C8)*

2.4 To use the immediate effects of exercise

In some cases, contact persons initiated exercise as a method with the stated purpose of lowering a patient's stress level in acute situations. A patient's mood affects other patients, so individual stress levels may affect the psychosocial climate throughout the forensic psychiatric care ward.

*(C9)*

3. The work with the patients' exercise is governed by different conditions

Different conditions govern the contact persons’ work with exercise. Some conditions were described as natural parts of the forensic psychiatric care context, while others were described as non-optimal or as direct obstacles to the contact persons' work with exercise. The conditions were divided into four subcategories (Table I).

3.1 The patients' individual conditions for exercise

The patients' individual conditions, which can apply to different areas, played a role in their ability to exercise. Participants noted that motivation varies between patients, ranging from early commitment and willingness to low motivation, although some form of interest was usually present. Furthermore, patients' mental states or psychiatric conditions can impact their ability to exercise.

*(C10)*

A patient's social ability or preferences were also noted as crucial conditions to consider. According to the participants, some patients were helped by group exercise and were therefore happy to participate in team sports. Such patients may have more difficulty with physical exercise that is conducted individually, for example, following an individual exercise program at the gym. Other patients were described as unable to perform exercise with others. An individual approach might be necessary to increase the opportunity for exercise for these patients.

3.2 The types of exercise available at the hospital

According to participants, the most important prerequisite for successful exercise was the availability of extensive and varied types of exercise activities. This allowed consideration of patients' preferences and conditions, which can increase the chance of finding a suitable form of exercise.

*(C11)*

The different available exercise options were described as an integral part of the hospital's operations. The exercise was scheduled and regularly recurring every week.

Participants had ideas for increasing the range of exercise activities present at the hospital to include more patients. One suggestion was to make better use of the outdoor environment by ensuring that there was equipment for ball games. Another was to supplement the offered exercise activities to better suit female patients, for example dance was suggested. Furthermore, already available activities, like the gym, could be offered for longer periods and during other times, for example, in the evening.

3.3 Organizational obstacles to working with patients' exercise

Some tasks and responsibilities of the contact persons were described as hindering work with patients' exercise. When local routines stipulated that patients should be followed by HCWs when leaving the ward, the remaining number of HCWs on the ward unit was consequently reduced. The number of remaining HCWs on the ward may then be too few to accompany patients to physical exercise according to rules for the minimum number of staff required on the ward. Exercise then risks being down-prioritized. If accompanying HCWs had not always been required when a patient was to leave the ward unit, for example, in the hospital's enclosed courtyard or to the exercise facilities in the hospital area, remaining patients may have more opportunities to be physically active.

*(C12)*

Similarly, there were always fewer HCWs on the ward in the evening than during the day, thereby limiting the opportunities for spontaneous exercise in the evening. Participants objected to this situation, wishing for a change.

Beyond the need for accompanying HCWs for patient exercise, certain safety routines were perceived as counterproductive for the work with patients’ exercise. One safety routine stipulated that patients should be searched and required to change clothes after being outside the ward, for example, after walking. Participants worried that patients may experience this as either offensive or too laborious. Instead, patients may choose to not go out at all and thus become less physically active.

*(C13)*

3.4 The low status of exercise as a treatment option

Although participants recognized that exercise was important for patients’ mood and well-being, they experienced only irregular and limited use of exercise for therapeutic purposes. Furthermore, different wards had different practices. Exercise was irregularly discussed and documented in patients’ treatment plans so that goals and purposes of the exercise were clear. Additionally, exercise was not necessarily linked to patients' mental states in the treatment plan. According to participants, this could be explained by exercise having a low status as a treatment option. The contact persons wanted the status of exercise to increase. Several ways of achieving this were described, such as an increased presence of HCWs with exercise expertise and an increased documentation of exercise in formal treatment plans. This would emphasize that exercise could be used as a treatment option for mental conditions such as anxiety, depression, or aggressive behavior.

*(C14)*

## Discussion

The results showed that the thoughts, notions and experiences of exercise among contact persons within inpatient forensic psychiatric care could be expressed under three qualitatively separated categories, *Exercise is meaningful to the patients, Different professional roles related to the patients' exercise,* and *The work with patients' exercise is governed by different conditions*.

### Awareness of the positive impact of physical activity on somatic and mental health

Study participants shared both detailed thoughts and extensive experiences regarding exercise in the context of forensic psychiatric care. They were generally positive about exercise and offered suggestions to increase exercise opportunities for patients. However, exclusively psychological and psychosocial effects were described and there was a lack of descriptions of physical exercise having effects within traditional target areas, such as muscle strength, oxygen uptake capacity, or weight control. Since patients in forensic psychiatric care often have complex psychiatric (Palijan et al., 2009), somatic comorbidities (Jackson et al., 2020; Launders et al., 2022) and low physical fitness (Bergman et al., 2020), a broad range of indications for exercise was present.

Participants reported rare formal use of PA in care plans, except to treat obesity. The International Organization of Physical Therapists in Mental Health has suggested that physical training should be part of the multiprofessional treatment work within psychiatric health care (Vancampfort et al., 2012). Obstacles to physical exercise should be addressed through collaborative efforts from the entire multiprofessional rehabilitation team. Exercise should be tailored to individual needs based on psychiatric symptoms, physical comorbidity, and side effects from pharmaceutical treatments. By applying a person-centered care perspective, individualized rehabilitation based on patient participation with shared decision making and common agreements, could be achieved (Ekman et al., 2011). The individualized perspective is supported by a recent systematic review (Hellzén et al., 2023).

The results presented herein may be seen as unveiling a somewhat ambiguous view on exercise within the forensic psychiatric care context. According to study participants, exercise was meaningful for patients, positively affected mood and well-being, and could be used for social training to improve everyday life skills. Still, exercise was perceived as having low status when it came to the planning of a patient’s treatment or rehabilitation. A national survey (Bergman et al., 2021) similarly showed that although it was common to prescribe exercise to patients in forensic psychiatric care, this was mostly done in a non-formal manner with no previous assessment and seldom to address treatment goals. This is in contrast to research describing numerous indications for exercise in patients with psychotic disorders (Jackson et al., 2020; Launders et al., 2022). According to recommendations for exercise in severe mental illness (Schuch et al., 2016), exercise should be included in the care plan in the same way as other pharmacological and non-pharmacological interventions within the forensic psychiatric rehabilitation. Since PA can improve physical fitness, physical function, and co-morbidity disorders like cardiovascular risk factors, psychiatric symptoms, depression and neurocognition (Firth et al., 2015; Knapen et al., 2015; Oertel-Knochel et al., 2014), it is a treatment option that may currently be underutilized in forensic psychiatric care.

### Contextual organizational or intrapersonal barriers

The participants described encouraging exercise as being part of their role as contact persons. This contrasts somewhat with the findings of a similar study (Kinnafick et al., 2018).

A survey of physiotherapists (Soundy et al., 2014) reported that they viewed lack of priority by other HCWs to be an important barrier to PA in mental health settings. Even if the participants in the present study did not express any lack of priority regarding exercise, they acknowledged that this could be the case among some of their colleagues, and that patients’ access to exercise could be dependent on this. Individually adapted support has been shown to positively affect patients’ adherence to exercise and lead to better results (Beebe et al., 2011, Mason & Holt, 2012, Soundy et al., 2014) when led by exercise experts (Stubbs et al., 2018, Vancampfort et al., 2016). An ambiguity might be reflected in the participants’ description of their professional roles related to patients’ exercise. On one hand, several professional roles were described by participants, however, participants also described that exercise was not a recognized area of responsibility, but rather something that some contact persons did if they had a special interest in exercise. Therefore, whether patients were supported by their contact person to exercise was considered a matter of chance.

A study from secure settings reported exercise as excluded from treatment plans (Kinnafick et al., 2018). This was to a certain degree confirmed herein, as well as the low status of exercise as an intervention. Participants also described benefits of cooperation with other categories of HCWs, such as physiotherapists, and proposed that increased participation of physiotherapists would be beneficial for increasing the status of exercise as a treatment option.

The fact that patients are confined in forensic psychiatric care presents a unique barrier to physical exercise. Participants expressed dissatisfaction with organizational barriers at the clinic that reduced their ability to promote patients’ PA. Even a patient who wanted and was able to exercise regularly did not necessarily have the opportunity to do so. Although it was shown in another study where the current hospital was included [Olausson et al., 2021] that even if an organization and environment supports the use of PA and exercise in care, there are still barriers due to ward rules. For example, patients must be accompanied by HCWs when leaving the units to go into the outdoor area, which may lead to staffing issues related to ward safety. The organization may provide fewer possibilities for spontaneous PA today compared to before, while the opportunities for organized physical exercise may have increased. Barriers described included relatively easily remediable organizational factors in the form of visitation rules and premise design.

Kinnafick et al. (2018) discussed alternative strategies to achieve increased PA within forensic psychiatric care. One suggestion was “informal strategies” such as informational and social support, another “compulsory intervention” such as compulsory exercise sessions. We would rather suggest “individual adaptation within the framework of team-based multimodal rehabilitation, after careful examination of conditions”.

### Strengths and limitations

When this study was initiated, research on PA within forensic psychiatric care and rehabilitation was limited. There was not enough knowledge in the field to investigate a “phenomenon”. A content analysis to describe critical areas was therefore chosen. A strength is that focus groups were used to facilitate a rich data collection (Kitzinger, 1995). Focus group members can offer each other support but also challenge each other’s views, which can lead to deeper insights (Kitzinger, 1995; Krueger, 2015). Another strength was that the participants were well-experienced in the field. They knew each other and worked in the same hospital with similar professional roles. However, the groups consisted of HCWs from different wards to be able to identify any differences in working methods.

Content analysis can be said to be based on a common-sense notion that we are capable of extracting meaning from what is said and that we understand what is meant to be said. To ensure confirmability, the discussions around themes were held within the whole author group. However, four of the authors (TN, PA, AD, RT) neither participated in the implementation of the focus group discussions, nor listened to the recorded data but relied on the written text. Nevertheless, the subcategories that emerged from our data may not constitute all possible subcategories within each respective category. It cannot be excluded that the results would have been different if the research had been conducted by other researchers, as pre-understanding potentially influences the interview and the way narratives are interpreted. However, preunderstanding, in terms of knowledge and experience of the context, is important for a deeper understanding and interpretation of what is said (Nyström & Dahlberg, 2001).

To enhance trustworthiness of the study (Lincoln & Guba, 1985), the analysis procedures were thoroughly described and followed the methodology outlined by Graneheim and Lundman (2004). To increase dependability, an interview guide was developed and pilot tested. However, follow-up questions asked during the interviews varied due to their semi-structured format. To increase the credibility of data analysis, multiple researchers with different professional backgrounds, experiences and pre-understandings were involved (Lincoln & Guba, 1985). Author 7 systematically supervised all steps of the analysis with continuous discussion and reflection during the process. Different levels of abstraction and interpretation were applied during the analysis process. Quotations are frequently presented in the results to provide clarity and enhance the credibility of the findings.

The selection of participants also affects the transferability of the results (Lincoln & Guba, 1985). Detailed inclusion criteria and participant descriptions are provided to assist the reader in assessing transferability to their own context. The results of this study may be considered to be transferable to other forensic psychiatric care hospitals.

Several years of PA promotion (at the studied clinic) with exercise experts supporting staff at the premises and a large range of physical activities offered to patients, might have given a biased view. Despite this somewhat more supportive context compared to forensic psychiatric care wards in general, there were still apparent contextual and attitude barriers to exercise.

A limitation of this study can be that the focus groups were conducted nine years ago, which may affect the relevance and timeliness of the findings. However, Swedish forensic psychiatric care has not undergone any major organizational changes since the study was conducted. Nor have the interventions or guidelines for care changed. Low use of PA and exercise within forensic psychiatric care remains, also mirrored in that half of the patients rate their somatic health as unsatisfactory, and present with a BMI ≥ 30 in about half of all patients (SWEDISH NATIONAL FORENSIC PSYCHIATRIC REGISTER, 2024).

### Ethical considerations

There are no obvious risks for participants of the focus groups. However, the discussion may raise questions and concerns among individual participants about their own attitude to what is being discussed in relation to the positions of the rest of the group. In a broader perspective, physical activity may be underutilized as a form of treatment in forensic psychiatric care. There is an ethical problem if patients are given access to a treatment based on the HCWs personal perceptions and attitudes, since treatment should be evidence based.

## Conclusions

Among HCWs, specifically contact persons within forensic psychiatric care, there was awareness of the positive impact of exercise on somatic and mental health and exercise was considered meaningful to patients. The contact persons identified contextual organizational and intrapersonal barriers to patients' opportunities and abilities to PA and exercise.

This study can contribute to the understanding of the role of the HCWs in supporting exercise for (in)patients with severe psychiatric illness. The increased knowledge can contribute to the better use of exercise as a treatment alternative or complement in forensic psychiatric care.

### Relevance for clinical practice

We suggest that a close cooperation between exercise experts, such as physiotherapists, contact persons, and nurses, would be an effective way to implement exercise interventions in the forensic psychiatric care context. This suggestion also relies on the previous findings from this author group indicating that exercise is not used in a formal way in forensic psychiatric care [Bergman et al., 2021]. If the need for PA and exercise is not included in care plans, exercise risks being de-prioritized, and the needs remain invisible. A better use of PA and exercise in forensic psychiatric care has, for patients with severe psychiatric illness, the potential to improve physical status, reduce morbidity in cardiovascular disease, contribute to stress management and reduced depressive symptoms, and to improve cognitive function.

Considering organizational barriers, one can discuss whether existing nursing staff should be trained in PA and exercise, or rather that HCWs with expertise in PA, and exercise, should be hired or both. It seems reasonable to recommend that exercise should be as an integrated part of forensic psychiatric care as it is in other team-based rehabilitation fields such as cardiac, stroke, or geriatric rehabilitation.

## References

[cit0001] Andine, P., & Bergman, H. (2019). Focus on brain health to improve care, treatment, and rehabilitation in forensic psychiatry. *Frontiers in Psychiatry*, *10*, 840. 10.3389/fpsyt.2019.00840 31849721 PMC6901922

[cit0002] Andreasson, H., Nyman, M., Krona, H., Meyer, L., Anckarsater, H., Nilsson, T., & Hofvander, B. (2014). Predictors of length of stay in forensic psychiatry: The influence of perceived risk of violence. *International Journal of Law and Psychiatry (Elmsford, NY)*, *37*, 635–642. 10.1016/j.ijlp.2014.02.038 24631525

[cit0003] Beebe, L. H., Smith, K., Burk, R., Mcintyre, K., Dessieux, O., Tavakoli, A., Tennison, C., & Velligan, D. (2011). Effect of a motivational intervention on exercise behavior in persons with schizophrenia spectrum disorders. *Community Mental Health Journal*, *47*, 628–636. 10.1007/s10597-010-9363-8 21113661 PMC3135691

[cit0004] Bergman, H., Nilsson, T., Andine, P., Degl'innocenti, A., Thomee, R., & Gutke, A. (2021). The use of physical exercise in forensic psychiatric care in Sweden: A nationwide survey. *Journal of Mental Health*, *33*, 1–9. 10.1080/09638237.2021.1875406 33487094

[cit0005] Bergman H, P. T., Nilsson T, P., Andine P, M. D., Degl'innocenti A, P., Thomee R, P. T., & Gutke A, P. T. (2020). Physical performance and physical activity of patients under compulsory forensic psychiatric inpatient care. *Physiotherapy Theory and Practice*, *36*, 507–515. 10.1080/09593985.2018.1488320 29927674

[cit0006] Caspersen, C. J., Powell, K. E., & Christenson, G. M. (1985). Physical activity, exercise, and physical fitness: Definitions and distinctions for health-related research. *Public Health Reports*, *100*(2), 126–131. 3920711 PMC1424733

[cit0007] Dauwan, M., Begemann, M. J., Heringa, S. M., & Sommer, I. E. (2016). Exercise Improves clinical symptoms, quality of life, global functioning, and depression in schizophrenia: A systematic review and meta-analysis. *Schizophr Bull*, *42*, 588–599. 10.1093/schbul/sbv164 26547223 PMC4838091

[cit0008] Degl' Innocenti, A., Alexiou, E., Andine, P., Striskaite, J., & Nilsson, T. (2021). A register-based comparison study of Swedish patients in forensic psychiatric care 2010 and 2018. *International Journal of Law and Psychiatry (Elmsford, NY)*, *77*. 101715. 10.1016/j.ijlp.2021.101715 34052684

[cit0009] Degl' Innocenti, A., Hassing, L. B., Lindqvist, A. S., Andersson, H., Eriksson, L., Hanson, F. H., Moller, N., Nilsson, T., Hofvander, B., & Anckarsater, H. (2014). First report from the Swedish National Forensic Psychiatric Register (SNFPR). *International Journal of Law and Psychiatry (Elmsford, NY)*, *37*, 231–237. 10.1016/j.ijlp.2013.11.013 24295538

[cit0010] Ekman, I., Swedberg, K., Taft, C., Lindseth, A., Norberg, A., Brink, E., Carlsson, J., Dahlin-Ivanoff, S., Johansson, I. L., Kjellgren, K., Liden, E., Ohlen, J., Olsson, L. E., Rosen, H., Rydmark, M., & Sunnerhagen, K. S. (2011). Person-centered care--ready for prime time. *European Journal of Cardiovascular Nursing : Journal of the Working Group on Cardiovascular Nursing of the European Society of Cardiology*, *10*, 248–251. 10.1016/j.ejcnurse.2011.06.008 21764386

[cit0011] Fazel, S., Wolf, A., Fiminska, Z., & Larsson, H. (2016). Mortality, rehospitalisation and violent crime in forensic psychiatric patients discharged from hospital: Rates and risk factors. *PLoS One*, *11*, e0155906. 10.1371/journal.pone.0155906 27196309 PMC4873227

[cit0012] Firth, J., Cotter, J., Elliott, R., French, P., & Yung, A. R. (2015). A systematic review and meta-analysis of exercise interventions in schizophrenia patients. *Psychologie Medicale*, *45*, 1343–1361. 10.1017/S0033291714003110 25650668

[cit0013] Firth, J., Stubbs, B., Rosenbaum, S., Vancampfort, D., Malchow, B., Schuch, F., Elliott, R., Nuechterlein, K. H., & Yung, A. R. (2017). Aerobic exercise improves cognitive functioning in people with schizophrenia: A systematic review and meta-analysis. *Schizophr Bull*, *43*, 546–556. 10.1093/schbul/sbw115 27521348 PMC5464163

[cit0014] Graneheim, U. H., & Lundman, B. (2004). Qualitative content analysis in nursing research: Concepts, procedures and measures to achieve trustworthiness. *Nurse Education Today*, *24*, 105–112. 10.1016/j.nedt.2003.10.001 14769454

[cit0015] Graneheim, U. H., Lindgren, B. M., & Lundman, B. (2017). Methodological challenges in qualitative content analysis: A discussion paper. *Nurse Education Today*, *56*, 29–34. 10.1016/j.nedt.2017.06.002 28651100

[cit0016] Happell, B., Scott, D., & Platania-Phung, C. (2013). Nurse views on the cardiometabolic health nurse as an approach to improving the physical health of people with serious mental illness in Australia. *International journal of mental health nursing*, *22*, 418–429. 10.1111/j.1447-0349.2012.00892.x 23211091

[cit0017] Hassan, J., Shannon, S., Tully, M. A., Mccartan, C., Davidson, G., Bunn, R., & Breslin, G. (2022). Systematic review of physical activity interventions assessing physical and mental health outcomes on patients with severe mental illness (SMI) within secure forensic settings. *Journal of Psychiatric and Mental Health Nursing*, *29*, 630–646. 10.1111/jpm.12832 35426209 PMC9544360

[cit0018] Hellzén, O., Hammarström, L., Ekman, O., & Andreassen Devik, S. (2023). A meta-ethnographic review of forensic psychiatry inpatient care. nursing staff experiences of the nurse-patient encounter. *Issues in Mental Health Nursing*, *44*(12), 1226–1236. 10.1080/01612840.2023.2259997 37801705

[cit0019] Howner, K., Andine, P., Bertilsson, G., Hultcrantz, M., Lindstrom, E., Mowafi, F., Snellman, A., & Hofvander, B. (2018). Mapping systematic reviews on forensic psychiatric care: A systematic review identifying knowledge gaps. *Frontiers in Psychiatry*, *9*, 452. 10.3389/fpsyt.2018.00452 30319459 PMC6167556

[cit0020] Howner, K., Andine, P., Engberg, G., Ekstrom, E. H., Lindstrom, E., Nilsson, M., Radovic, S., & Hultcrantz, M. (2019). Pharmacological treatment in forensic psychiatry-a systematic review. *Frontiers in Psychiatry*, *10*, 963. 10.3389/fpsyt.2019.00963 32009993 PMC6976536

[cit0021] Jackson, C. A., Kerssens, J., Fleetwood, K., Smith, D. J., Mercer, S. W., & Wild, S. H. (2020). Incidence of ischaemic heart disease and stroke among people with psychiatric disorders: Retrospective cohort study. *British Journal of Psychiatry*, *217*, 442–449. 10.1192/bjp.2019.250 PMC7511900 31753047

[cit0022] Kazemi, A., Soltani, S., Aune, D., Hosseini, E., Mokhtari, Z., Hassanzadeh, Z., Jayedi, A., Pitanga, F., & Akhlaghi, M. (2024). Leisure-time and occupational physical activity and risk of cardiovascular disease incidence: A systematic-review and dose-response meta-analysis of prospective cohort studies. *International Journal of Behavioral Nutrition and Physical Activity*. *21*(1), 45. 10.1186/s12966-024-01593-8 38659024 PMC11044601

[cit0023] Kinnafick, F. E., Papathomas, A., & Regoczi, D. (2018). Promoting exercise behaviour in a secure mental health setting: Healthcare assistant perspectives. *International Journal of Mental Health Nursing*, *27*, 1776–1783. 10.1111/inm.12484 29847009

[cit0024] Kitzinger, J. (1995). Qualitative research. Introducing focus groups. *BMJ*, *311*, 299–302. 10.1136/bmj.311.7000.299 7633241 PMC2550365

[cit0025] Knapen, J., Vancampfort, D., Morien, Y., & Marchal, Y. (2015). Exercise therapy improves both mental and physical health in patients with major depression. *Disability and Rehabilitation*, *37*, 1490–1495. 10.3109/09638288.2014.972579 25342564

[cit0026] Krueger, R. A. (2015). R. A. Focus groups: A practical guide for applied research. 5. [updated] ed. In Casey, M. A. (Ed.) Thousand Oaks, CA: Sage Publications.

[cit0027] Launders, N., Kirsh, L., Osborn, D. P. J., & Hayes, J. F. (2022). The temporal relationship between severe mental illness diagnosis and chronic physical comorbidity: A UK primary care cohort study of disease burden over 10 years. *Lancet Psychiatry*, *9*, 725–735. 10.1016/S2215-0366(22)00225-5 35871794 PMC9630158

[cit0028] Lincoln, Y., & Guba, E. (1985). *Naturalistic inquiry* (1st ed.). Newbury Park, CA: Sage.

[cit0029] Mason, O. J., & Holt, R. (2012). Mental health and physical activity interventions: A review of the qualitative literature. *Journal of Mental Health*, *21*, 274–284. 10.3109/09638237.2011.648344 22533784

[cit0030] Nygard, M., Brobakken, M. F., Roel, R. B., Taylor, J. L., Reitan, S. K., Guzey, I. C., Morken, G., Vedul-Kjelsas, E., Wang, E., & Heggelund, J. (2019). Patients with schizophrenia have impaired muscle force-generating capacity and functional performance. *Scandinavian Journal of Medicine and Science in Sports*, *29*, 1968–1979. 10.1111/sms.13526 31359490

[cit0031] Nyström, M., & Dahlberg, K. (2001). Pre-understanding and openness – A relationship without hope? *Scandinavian Journal of Caring Sciences*, *15*(4), 339–346. 10.1046/j.1471-6712.2001.00043.x 12453176

[cit0032] Oertel-Knochel, V., Mehler, P., Thiel, C., Steinbrecher, K., Malchow, B., Tesky, V., Ademmer, K., Prvulovic, D., Banzer, W., Zopf, Y., Schmitt, A., & Hansel, F. (2014). Effects of aerobic exercise on cognitive performance and individual psychopathology in depressive and schizophrenia patients. *European Archives of Psychiatry and Clinical Neuroscience*, *264*, 589–604. 10.1007/s00406-014-0485-9 24487666

[cit0033] Olausson, S., Wijk, H., Johansson Berglund, I., Pihlgren, A., & Danielson, E. (2021). Patients' experiences of place and space after a relocation to evidence-based designed forensic psychiatric hospitals. *International journal of mental health nursing*, *30*, 1210–1220. 10.1111/inm.12871 33939249

[cit0034] Palijan, T. Z., Muzinic, L., & Radeljak, S. (2009). Psychiatric comorbidity in forensic psychiatry. *Psychiatria Danubina*, *21*, 429–436. 19794370

[cit0035] Pedersen, A. L. W., Lindekilde, C. R., Andersen, K., Hjorth, P., & Gildberg, F. A. (2021). Health behaviours of forensic mental health service users, in relation to smoking, alcohol consumption, dietary behaviours and physical activity-A mixed methods systematic review. *Journal of Psychiatric and Mental Health Nursing*, *28*, 444–461. 10.1111/jpm.12688 32916759

[cit0036] Reddigan, J. I., Ardern, C. I., Riddell, M. C., & Kuk, J. L. (2011). Relation of physical activity to cardiovascular disease mortality and the influence of cardiometabolic risk factors. *American Journal Of Cardiology*, *108*, 1426–1431. 10.1016/j.amjcard.2011.07.005 21855834

[cit0037] Sariaslan, A., Sharpe, M., Larsson, H., Wolf, A., Lichtenstein, P., & Fazel, S. (2022). Psychiatric comorbidity and risk of premature mortality and suicide among those with chronic respiratory diseases, cardiovascular diseases, and diabetes in Sweden: A nationwide matched cohort study of over 1 million patients and their unaffected siblings. *PLoS medicine*, *19*, e1003864. 10.1371/journal.pmed.1003864 35085232 PMC8794193

[cit0038] Schuch, F. B., Vancampfort, D., Richards, J., Rosenbaum, S., Ward, P. B., & Stubbs, B. (2016). Exercise as a treatment for depression: A meta-analysis adjusting for publication bias. *Journal of Psychiatric Research*, *77*, 42–51. 10.1016/j.jpsychires.2016.02.023 26978184

[cit0039] Shimada, T., Ito, S., Makabe, A., Yamanushi, A., Takenaka, A., Kawano, K., & Kobayashi, M. (2022). Aerobic exercise and cognitive functioning in schizophrenia: An updated systematic review and meta-analysis. *Psychiatry Research (Limerick)*, *314*. 114656. 10.1016/j.psychres.2022.114656 35659670

[cit0040] Sivak, L., Forsman, J., & Masterman, T. (2023). Duration of forensic psychiatric care and subsequent criminal recidivism in individuals sentenced in Sweden between 2009 and 2019. *Frontiers in Psychiatry*, *14*. 1129993. 10.3389/fpsyt.2023.1129993 37009123 PMC10053040

[cit0041] Soundy, A., Stubbs, B., Probst, M., Hemmings, L., & Vancampfort, D. (2014). Barriers to and facilitators of physical activity among persons with schizophrenia: A survey of physical therapists. *Psychiatric Services*, *65*, 693–696. 10.1176/appi.ps.201300276 24585134

[cit0042] Stanton, R., Happell, B., & Reaburn, P. (2015a). Investigating the exercise-prescription practices of nurses working in inpatient mental health settings. *International Journal of Mental Health Nursing*, *24*, 112–120. 10.1111/inm.12125 25639383

[cit0043] Stanton, R., Reaburn, P., & Happell, B. (2015b). Barriers to exercise prescription and participation in people with mental illness: The perspectives of nurses working in mental health. *Journal of Psychiatric and Mental Health Nursing*, *22*, 440–448. 10.1111/jpm.12205 25855247

[cit0044] Stubbs, B., Williams, J., Gaughran, F., & Craig, T. (2016). How sedentary are people with psychosis? A systematic review and meta-analysis. *Schizophrenia Research (Amsterdam)*, *171*, 103–109. 10.1016/j.schres.2016.01.034 26805414

[cit0045] Stubbs, B., Vancampfort, D., Hallgren, M., Firth, J., Veronese, N., Solmi, M., Brand, S., Cordes, J., Malchow, B., Gerber, M., Schmitt, A., Correll, C. U., De Hert, M., Gaughran, F., Schneider, F., Kinnafick, F., Falkai, P., Moller, H. J., & Kahl, K. G. (2018). EPA guidance on physical activity as a treatment for severe mental illness: A meta-review of the evidence and position statement from the european psychiatric association (EPA), supported by the international organization of physical therapists in mental health (IOPTMH). *European Psychiatry*, *54*, 124–144. 10.1016/j.eurpsy.2018.07.004 30257806

[cit0046] Svennerlind, C., Nilsson, T., Kerekes, N., Andine, P., Lagerkvist, M., Forsman, A., Anckarsater, H., & Malmgren, H. (2010). Mentally disordered criminal offenders in the Swedish criminal system. *International Journal of Law and Psychiatry (Elmsford, NY)*, *33*, 220–226. 10.1016/j.ijlp.2010.06.003 20667594

[cit0047] SWEDISH NATIONAL FORENSIC PSYCHIATRIC REGISTER, Rättspsy K. Annual report. (2024). Gothenburg: Swedish National Forensic Psychiatric Register.

[cit0048] Toots, A., Littbrand, H., Lindelof, N., Wiklund, R., Holmberg, H., Nordstrom, P., Lundin-Olsson, L., Gustafson, Y., & Rosendahl, E. (2016). Effects of a high-intensity functional exercise program on dependence in activities of daily living and balance in older adults with dementia. *Journal of the American Geriatrics Society*, *64*, 55–64. 10.1111/jgs.13880 26782852 PMC4722852

[cit0049] Ulrich, R., Bogren, L. K., Gardiner, S. K., & &amp, S. (2018). Psychiatric ward design can reduce aggressive behavior. *J Environmental Psychology*, *57*, 53–66. 10.1016/j.jenvp.2018.05.002

[cit0050] Vancampfort, D., Rosenbaum, S., Schuch, F. B., Ward, P. B., Probst, M., & Stubbs, B. (2016). Prevalence and predictors of treatment dropout from physical activity interventions in schizophrenia: A meta-analysis. *General Hospital Psychiatry*, *39*, 15–23. 10.1016/j.genhosppsych.2015.11.008 26719106

[cit0051] Vancampfort, D., Rosenbaum, S., Schuch, F., Ward, P. B., Richards, J., Mugisha, J., Probst, M., & Stubbs, B. (2017). Cardiorespiratory fitness in severe mental illness: A systematic review and meta-analysis. *Sports Medicine*, *47*, 343–352. 10.1007/s40279-016-0574-1 27299747

[cit0052] Vancampfort, D, De Hert, M, Skjerven, L. H, Gyllensten, A. L, Parker, A, Mulders, N, Nyboe, L, Spencer, F, & Probst, M (2012). International organization of physical therapy in mental health consensus on physical activity within multidisciplinary rehabilitation programmes for minimising cardio-metabolic risk in patients with schizophrenia. *Disability and Rehabilitation*, *34*, 1–12. 10.3109/09638288.2011.587090 21957908

[cit0053] Wynaden, D., Barr, L., Omari, O., & Fulton, A. (2012). Evaluation of service users’ experiences of participating in an exercise programme at the western australian state forensic mental health services. *International Journal of Mental Health Nursing*, *21*, 229–235. 10.1111/j.1447-0349.2011.00787.x 22533330

[cit0054] Zschucke, E., Heinz, A., & Strohle, A. (2012). Exercise and physical activity in the therapy of substance use disorders. *The Scientific World Journal*, *2012*, 901741. 10.1100/2012/901741 22629222 PMC3354725

